# Hypospadias: clinical approach, surgical technique and long-term outcome

**DOI:** 10.1186/s12887-021-02941-4

**Published:** 2021-11-26

**Authors:** Pier Luca Ceccarelli, Laura Lucaccioni, Francesca Poluzzi, Anastasia Bianchini, Diego Biondini, Lorenzo Iughetti, Barbara Predieri

**Affiliations:** 1 Pediatric Surgical Unit, Department of Medical and Surgical Sciences for Mothers, Children and Adults, University of Modena and Reggio Emilia, via del Pozzo 71, 41124 Modena, Italy; 2 Pediatric Unit, Department of Medical and Surgical Sciences of the Mothers, Children and Adults, University of Modena and Reggio Emilia, via del Pozzo 71, 41124 Modena, Italy

**Keywords:** Hypospadias, Long term outcome, Surgery

## Abstract

**Background:**

Hypospadias is one of the most common congenital abnormalities in male newborn. There is no universal approach to hypospadias surgical repair, with more than 300 corrective procedures described in current literature. The reoperation rate within 6–12 months of the initial surgery is most frequently used as an outcome measure. These short-term outcomes may not reflect those encountered in adolescence and adult life. This study aims to identify the long-term cosmetic, functional and psychosexual outcomes.

**Methods:**

Medical records of boys who had undergone surgical repair of hypospadias by a single surgical team led by the same surgeon at a single centre between August 2001 and December 2017 were reviewed. Families were contacted by telephone and invited to participate. Surgical outcome was assessed by combination of clinical examination, a life-related interview and 3 validated questionnaires (the Penile Perception Score-PPS, the Hypospadias Objective Score Evaluation-HOSE, the International Index of Erectile Function-5-IIEF5). Outcomes were compared according to age, severity of hypospadias, and respondent (child, parent and surgeon).

**Results:**

187 children and their families agreed to participate in the study. 46 patients (24.6%) presented at least one complication after the repair, with a median elapsed time of 11.5 months (6.5–22.5). Longitudinal differences in surgical corrective procedures (*p* < 0.01), clinical approach (p < 0.01), hospitalisation after surgery (p < 0.01) were found. Cosmetic data from the PPS were similar among children and parents, with no significant differences in child’s age or the type of hypospadias: 83% of children and 87% of parents were satisfied with the cosmetic result. A significant difference in functional outcome related to the type of hypospadias was reflected responses to HOSE amongst all groups of respondents: children (*p* < 0.001), parents (p=0.02) and surgeon (*p* < 0.01). The child’s HOSE total score was consistently lower than the surgeon (p < 0.01). The HOSE satisfaction rate on functional outcome was 89% for child and 92% for parent respondents.

**Conclusion:**

Surgeons and clinicians should be cognizant of the long-term outcomes following hypospadias surgical repair and this should be reflected in a demand for a standardised approach to repair and follow-up.

## Introduction

Hypospadias is one of the commonest congenital malformations affecting the penis, with a reported incidence of 1/250 newborns, and yet unknown etiology [1]. Three typical anatomical features define hypospadias: ectopic location of the urethral meatus, irregular distribution of the foreskin and abnormal ventral curvature of the penis. It appears that a universal approach to hypospadias surgical repair is missing, with more than 300 corrective procedures described in the current literature [2–4]. The reoperation rate, within 6–12 months since the initial surgery, is most frequently used as an outcome measure. The complication rate, generally estimated within the short term, has been reported as 5–10% for mild forms and 15–56% for severe forms of hypospadias [5]. These short-term outcome measures are inadequate and may not reflect the experience of boys in puberty and beyond. A reliable final outcome of the repair could not be predicted within a 12-month follow-up of surgery, as psychosexual development is not complete and pubertal physical changes may affect the final cosmetic appearance and function of the corrected penis [6, 7]. Unfortunately, longitudinal studies following hypospadias repair are still rare and the real impact in adolescence and adult life remains uncertain. This may be due to lack of condition specific validated patient-related outcome (PRO) measures [8] and objective tools to evaluate young people and families [9]. Reported outcomes in literature to date have been contradictory with comparison between reports complicated by the lack of consensus on the optimal surgical approach and follow-up [6, 10–16].

The aim of our study was to identify the long-term cosmetic, functional and psychosexual outcomes in boys who have previously undergone surgical repair for hypospadias from a single center.

## Materials and methods

This is a cross-sectional study medical and surgical records of children treated for hypospadias at the Pediatric Surgery Unit of Modena University Hospital by a single surgical team led by the same surgeon between August 1st, 2001 and December 31st, 2017 were reviewed. Boys, of any age, were included to whom a diagnosis of hypospadias had been made (all severities), if a minimum period of 6 months follow up after the initial surgical repair was completed. Three time periods (2001–2006, 2007–2012, 2013–2017) were compared to evaluate variations in clinical and surgical approach.

Families were initially approached by telephone and invited to participate. Families wishing to participate but unable to attend the clinical evaluation received questionnaires by e-mail. The consultation was made by the same surgeon who had performed the surgery, after at least 12 months from the surgical repair, and an identification number was given to each child/family: responses were collected from children, parent/guardian and surgeon, using three anonymous validated questionnaires.

The Hypospadias Objective Scoring Evaluation (HOSE) [17] is used for assessing functional outcomes after the corrective surgery; a score ≥ 14 points is defined as normal for the population. HOSE was administered to those children older than 10 years-old at the time of baseline visit, as well as to their parent/guardian and surgeon. The Pediatric Penile Perception Score (PPPS) [18] is designed to evaluate the cosmetic outcome following surgical repair; a score of ≥12 points is defined as normal for the population. PPPS was administered to those children older than 10 years-old at the time of baseline visit and to their parent/guardian. Finally, the International Index of Erectile Function 5 (IIEF-5) [19] analyses the sexual activity of subjects with or without erectile dysfunction (ED) was administered to young men older than 18 years-old who had undergone previous hypospadias repair.

The boys/young men and their parents filled the forms independently. An interviewer was available should either party require assistance. The surgeon was asked to complete the HOSE immediately after each clinical follow-up evaluation.

Data were checked for normal distribution using the Kolmogorov–Smirnov statistics, and nonparametric statistical analysis (IBM SPSS Statistics software, Version 20, San Francisco, USA) was performed. Data are expressed as median (interquartile) and percentages. Chi-squared tests were used to compare discrete variables, while between-group comparisons of continuous variables were evaluated using Mann–Whitney’s U-test and Kruskal–Wallis when appropriate. Statistical significance was set at *p* value < 0.05. The study was approved by the Local Ethics Committee (EC 400/17).

## Results

### Population of the study – baseline characteristics

Three hundred ninety-eight children met our inclusion criteria and were approached. One hundred ninety-four families agreed to participate, and of the 11 who only to completed questionnaires without a corresponding clinical evaluation, 4 returned completed. The remaining 183 families consented to both clinical evaluation and completion of questionnaires. The response rate was 47%, with no significant differences in disease severity (p=0.32) or short-term complication frequency (p=0.20) amongst respondents and non-respondents.

Table 1 summarizes clinical history of the study population extrapolated from their medical and surgical records.

**Table 1:** Baseline characteristics of the study population (187 patients)

Median age at time of surgery (interquartile)	2 years (1–3)
Born:	
- preterm (GA < 37 weeks)	32 (17.1%)
- at term (GA > 37 weeks)	155 (82.9%)
Type of hypospadias at presentation^a^:	
- Distal	168 (90.3%)
- Mid-shaft	6 (3.2%)
- Proximal	12 (6.4%)
Anomalies associated at hypospadias:	
- Undescended testis	10 (5.4%)
- Inguinal hernia	7 (3.7%)
- Both	12 (6.4%)

^a^Data was missing for one patient

### Surgical approaches and temporal differences

Four surgical approaches were used: the MAGPI (Meatal Advancement and Glanuloplasty Procedure Incorporated) procedure, the TIP (Tubularized Incised Plate) urethroplasty with or without the Retik variation, and the Duckett technique [2]. Except for one patient, whose data were missing, the MAGPI was performed in 40 boys (21.3%), the TIP in 125 boys (67.4%) [with the Retik variation in 60 (32%) of them], and the Duckett in 21 boys (11.2%).

The three time periods of the study were compared to evaluate for variation in clinical and surgical approach. Table 2 summarizes the number of patients who underwent surgery for hypospadias according to each surgical technique. Longitudinal differences in age at first surgical intervention (*p* = 0.028), type of surgical corrective procedure utilized (*p* < 0.01), clinical approach after surgery (duration of antibiotic treatment, duration of urethral catheterization, kind of medication) (p < 0.01, p=0.02, p=0.023, respectively) and length of hospitalization (p < 0.01) were found between the three temporal groups.

**Table 2:** The number of patients who underwent surgery for hypospadias along time, divided for surgical technique in the three different times of the study

	2000–2006	2007–2021	2013–2017	TOTAL
**MAGPI**	8	20	12	40
**TIP (Retik)**	39	50 (25)	36 (35)	125 (60)
**Dukett**	1	11	9	21
**TOTAL**	48	81	57	

### Complications

The median age at the time of the first clinical assessment following surgical procedure was 14 days (range 8-20). Out of the whole population, forty-six boys (24.6%) presented with at least one complication following their initial repair, with a median elapsed time of 11.5 months (range 6.5–22.5).

Surgical complications included: 19 fistulas (10.2%), 15 stenosis (8%), 6 dehiscence (3.2%), 6 other or mixed complications (3.2%). The incidence of complication has changed over the study period, and an overall decrease has been registered (*p* = 0.047).

### Satisfaction rate for functional outcomes

The satisfaction rate for functional outcomes was assessed in 91 patients. The HOSE scores exceeded the score of 14 in 89% of the boys’ (81/91), 92% of parents’ (167/180) and 96% of the surgeons’ evaluations (176/182) (Table 3). The boys’ HOSE total score was consistently lower than the surgeon’s one (*p* < 0.01).

**Table 3:** How patients, parents and surgeon scored HOSE and PPS questionnaire

		2001–2006		2007–2012		2013–2017		Total
		≥14	< 14	≥14	< 14	≥14	< 14	
HOSE	Patients	42	7	36	3	3	0	91
	Parents	41	4	75	5	49	4	180
	Surgeon	46	2	78	1	49	3	182
		≥12	< 12	≥12	< 12	≥12	< 12	
PPS	Patients	41	8	33	7	4	0	93
	Parents	39	7	67	11	52	5	181

HOSE scores were then analyzed in relation to the severity of hypospadias and to the child’s age at the time of evaluation (< 14 years versus ≥14 years).

A significant difference in functional outcome relating to the severity of hypospadias was reflected in the HOSE total scores in all groups: boys (*p* < 0.001), parents (*p* = 0.02) and surgeon (p < 0.01). All three groups scored consistently lower in severe hypospadias (proximal hypospadias).

No significant differences in the HOSE scores were found for the three groups in relation to the current age of the child.

### Satisfaction rate for cosmetic outcomes

The satisfaction rate for cosmetic outcome was assessed in 93 patients. Longitudinal cosmetic data from the PPPS between children and their parents were similar. PPPS total score was found to be greater than 12 in 78/93 (83%) of the boys and in 158/181 (87%) of the parents who responded (Table 3).

PPPS scores were then analyzed according to the age of the child at the time of evaluation (< 14 years versus ≥14 years). No differences were found in PPPS total scores according to age, in both parents and child subject groups (*p* = 0.1 and p = 0.1, respectively).

There were no differences in total PPPS scores between child and parent subject groups when analyzed according to severity of hypospadias (p = 0.1 and p = 0.1, respectively).

### Satisfaction rate for erectile function

Nine young men were over 18 years old at the time of evaluation. Four out of 9 men had previous sexual intercourse and were able to complete the IIEF-5 questionnaire, scoring 18, 22, 24 and 25.

## Discussion

This study evaluated long-term clinical, cosmetic and functional outcomes in hypospadias, and pointed out the necessity for clinicians and surgeons to be cognizant of the long-term clinical and functional complications, which may arise following surgical repair of hypospadias [20, 21]. They should design services which allow the necessary follow-up during adolescence and adult life. Recommendations to continue follow-up until pubertal onset clearly established had gone unheard, with most emphasis focused only on short-term outcomes in the first 12 months following initial surgery. Previous studies reporting on the opinions of young men and their families have unfortunately not culminated in the necessary change in approach both in standardization of the initial surgical technique and long-term follow-up [22–24].

Reports of previous studies have at times appeared contradictory and been criticized for having small or biased samples, without long-term follow-up and poor response rates thus limiting their applicability and the influence they might have on clinical care. The present study obtained a response rate of 47%, with no difference in disease severity or short-term complication frequency amongst respondents and non-respondents. More recently, literature is focusing on long term functional and cosmetic outcomes after surgery for hypospadias [25–28].

Data presented here show an improvement in surgical outcome over the study period with a decrease in frequency of complication between 2001 and 2017, which may relate to the relative experience of the surgical team conducting the repairs [29]. In addition, authors would suggest that such data on surgical outcomes corroborate those in current literature, and strongly advises that surgeons master fewer surgical techniques to improve outcomes [30]. The evolution of surgical practice for proximal forms of hypospadias is similarly reflected in our data with a trend towards a one-stage procedure rather than a two-stage one [31].

In this study functional outcomes have been collected using the HOSE evaluation tool, provided to child, parents and surgeon. The HOSE data showed a satisfaction rate higher than previously reported in literature following hypospadias repair (50–70%) [32]. Differences in HOSE scores have been found according to the severity of hypospadias in each of the subject groups (child, parent and surgeon) with lowest scores for functional outcomes in severe hypospadias.

It is of interest that child’s HOSE score was notably lower than that of the surgeon. The disparity between the opinion of the patient and surgeon following hypospadias repair has been documented widely in previous reports, perhaps stressing the need for an objective evaluation by an un-involved surgeon in patient’s care, to prevent bias in outcomes’ assessment [33]. Findings from this study support this statement, as the surgeon’s point of view is sensibly more optimistic in the functional outcomes’ evaluation than the patients’ [34].

In contrast, children’s and parents’ responses for cosmetic outcomes using PPPS had been found similar, neither influenced by age of child or the severity of hypospadias illustrating the consistency within families and the reliability of self-evaluation. The reported high satisfaction rate, in both children and parents, is similar to that of previous reports [9, 12], suggesting that families are happy with the appearance of the genitalia and that hypospadias is not associated with a negative self-perception.

It has been chosen to collect the opinion of parents in addition to those of the child, as authors believe it is essential to include the family as a whole when considering long-term outcomes of surgeries spanning childhood, adolescence and adulthood.

The limited numbers of young men that responded to the questionnaire relating to sexual function made the authors not able to infer any long-term outcomes relating to sexual function. It is expected to encourage clinicians to collect data regarding sexual function in future long-term follow-up studies.

The present study supports the need for a standardized evaluation of the long-term outcomes after hypospadias repair. However, findings demand the development of validated and condition specific questionnaires in order to confirm the findings.

We are conscious that the study presents some limitations. One of them is the response rate. In fact, our population response rate was lower that 50%, although from a very long follow up. Moreover, in our population we have a low percentage of young adults that could have been useful to increase the sexual outcomes. Moreover a control group is missing.

## Conclusions

The optimal method to assess the long-term outcome of hypospadias surgical repair is not yet established. Results suggest that the adoption of a combined evaluation, with input from child, parents and surgeons, can provide valuable insight into the long-term outcome in adolescence and early adulthood. Understanding the perspective of families on long-term outcomes following surgical repair might result in improvements in patient care.

## Data Availability

The data analyzed in this study is subject to the following licenses/restrictions: Datasets consist of data routinely recorded in clinical practice and anonymously recorded. Requests to access these datasets should be directed to Prof. Iughetti Lorenzo, iughetti.lorenzo@unimore.it.

## Abbreviations

ED: Erectile Dysfunction
HOSE: Hypospadias Objective Score Evaluation
IIEF5: International Index of Erectile Function-5
MAGPI: Meatal Advancement and Glanuloplasty Procedure Incorporated
PPS: Penile Perception Score
PRO: Patient Related Outcome
TIP: Tubularized Incised Plate
